# Cross-cultural translation, adaptation and validation of a Japanese version of the functional index for hand osteoarthritis (J-FIHOA)

**DOI:** 10.1186/s12891-020-03193-6

**Published:** 2020-03-16

**Authors:** Yasunobu Nakagawa, Shigeru Kurimoto, Emmanuel Maheu, Yuichiro Matsui, Yuri Kanno, Kunitaka Menuki, Masanori Hayashi, Tetsuya Nemoto, Takanobu Nishizuka, Masahiro Tatebe, Michiro Yamamoto, Katsuyuki Iwatsuki, Renée Liliane Dreiser, Hitoshi Hirata

**Affiliations:** 1 Department of Hand Surgery, Nagoya University Graduate School of Medicine, 65 Tsurumai-cho, Showa-ku, Nagoya, 466-8550 Japan; 2 Department of Rheumatology, AP-HP, Hospital Saint-Antoine, Paris, France; 3 Department of Orthopaedic Surgery, Faculty of Medicine and Graduate School of Medicine, Hokkaido University, Sapporo, Japan; 4 Hand Surgery and Microsurgery Center, Yotsuya Medical Cube, Tokyo, Japan; 5 Department of Orthopaedic Surgery, School of Medicine, University of Occupational and Environmental Health, Kitakyushu, Japan; 6 Department of Orthopaedic Surgery, Shinshu University School of Medicine, Matsumoto, Japan; 7 Department of Orthopaedic Surgery, Showa University School of Medicine, Tokyo, Japan; 8 Nagoya Hand Surgery Center, Chunichi Hospital, Nagoya, Japan; 9 Department of Rheumatology, AP-HP, Hospital Bichât, Paris, France

**Keywords:** Hand osteoarthritis, Translation, FIHOA, Japan, Evaluation study, Patient reported outcome measure

## Abstract

**Background:**

Hand osteoarthritis (OA) has a wide spectrum of clinical presentations and physical function is one of the core domains where patients suffer. The Functional Index for Hand Osteoarthritis (FIHOA) is a leading assessment tool for hand OA-related functional impairment. Our objective was to make a Japanese version of FIHOA (J-FIHOA) and validate it among Japanese hand OA patients.

**Methods:**

Forward and backward translation processes were completed to create a culturally adapted J-FIHOA. A prospective, observational multicenter study was undertaken for the validation process. Seventeen collaborating hospitals recruited Japanese hand OA patients who met the American College of Rheumatology criteria. A medical record review and responses to the following patient-rated questionnaires were collected: J-FIHOA, Hand20, Health Assessment Questionnaire (HAQ), numerical rating scale for pain (NRS pain) and Short Form 36 Health Survey (SF-36). We explored the structure of J-FIHOA using factor analysis. Cronbach’s alpha coefficients and item-total correlations were calculated. Correlations between J-FIHOA and other questionnaires were evaluated for construct validity. Participants in clinically stable conditions repeated J-FIHOA at a one- to two-week interval to assess test-retest reliability. To evaluate responsiveness, symptomatic patients who started new pharmacological treatments had a 1-month follow-up visit and completed the questionnaires twice. Effect size (ES) and standardized response mean (SRM) were calculated with pre- and post-treatment data sets. We assessed responsiveness, comparing ES and SRM of J-FIHOA with other questionnaires (construct approach).

**Results:**

A total of 210 patients participated. J-FIHOA had unidimensional structure. Cronbach’s alphas (0.914 among females and 0.929 among males) and item-total correlations (range, 0.508 to 0.881) revealed high internal consistency. Hand20, which measures upper extremity disability, was strongly correlated with J-FIHOA (*r* = 0.82) while the mental and role-social components of SF-36 showed no correlations (*r* = − 0.24 and − 0.26, respectively). Intraclass correlation coefficient for test-retest reliability was 0.83 and satisfactory. J-FIHOA showed the highest ES and SRM (− 0.68 and − 0.62, respectively) among all questionnaires, except for NRS pain.

**Conclusions:**

Our results showed J-FIHOA had good measurement properties to assess physical function in Japanese hand OA patients both for ambulatory follow-up in clinical practice, and clinical research and therapeutic trials.

## Background

Hand osteoarthritis (OA) is a highly prevalent and heterogeneous musculoskeletal disorder [1]. Aging increases the risk of its emergence and progression [2]. These issues are especially the case in Japan with its elderly population dramatically growing. While racial differences and gene variants are reported to influence the development of OA, comparatively few studies concern Asian countries [3, 4]. Epidemiologically, the prevalence in Asians such as Japanese and Chinese has been reported to be lower than in white U.S. individuals [5, 6]. Yet, a recent study showed a much higher prevalence of radiographic hand OA in the Japanese population compared to other cohorts [7–9]. These inconsistencies may be precipitated by heterogeneous presentations and limit the advancement of our understanding of this disease.

Although most Asian studies have focused on radiographic changes of the patients, not all structural damage manifests hand pain or stiffness [10]. A discrepancy exists between the radiographic findings and severity of symptoms [11]. Clinical courses differ in severity, number and distribution of symptomatic joints. There are various hand OA phenotypes that may impact the different outcomes in this disease. Certain patients experience a wide range of deteriorating health conditions, even beyond those with rheumatoid arthritis (RA) [12, 13]. In addition, some symptoms worsen longitudinally while others improve spontaneously [14]. Physicians and researchers need to distinguish symptomatic from non-symptomatic hand OA patients to both pursue appropriate treatment and engage in future clinical investigations.

Patient reported outcome measures (PROMs) generally play a key role when assessing arthritic conditions such as pain [15], physical disability [16], psychiatric disturbance [17], aesthetic problems [18, 19] and poor health-related quality of life [12, 13]. While a growing number of instruments are available and cover a wide range of the health sciences, we must use those most appropriate to the clinical setting [20, 21]. Since Japan is linguistically not Indo-European and culturally East Asian, we have to consider these differences when introducing new PROMs, many of which were developed in the West [22, 23].

Physical function is one of the core domains in which hand OA patients suffer. The Functional Index for Hand Osteoarthritis (FIHOA) was developed by Dreiser and Maheu in the 1990s and has been a widely adopted PROM in hand OA research [24, 25]. It assesses hand OA-specific disability utilizing a 10 question, 4-point Likert scale (from possible without difficulty to impossible) and has good measurement properties [24–27]. The total score ranges from 0 to 30. A score of at least 5 points has been validated as able to discriminate symptomatic from non-symptomatic hand OA [24]. Approximately 20 linguistic versions are available and others are being developed [28–31]. The original English version and relevant references are freely accessible at FIHOA.net. The FIHOA is recommended as an assessment tool for physical function by the Osteoarthritis Research Society International (OARSI) and European Society on Clinical and Economic Aspects of Osteoporosis, Osteoarthritis and Musculoskeletal Diseases (ESCEO) [32, 33]. Introducing the FIHOA to Japan will not only stimulate Japanese research but also add to the cumulative knowledge of hand OA.

The objective of this study was to create a Japanese version of the FIHOA (J-FIHOA) and to validate it among hand OA patients in Japan where few reliable, validated PROMs for hand OA are currently available.

## Methods

The original FIHOA assesses hand OA-related functional disability, consisting of 10 questions. We translated and cross-culturally adapted the FIHOA following established guidelines [22, 23]. Subsequently, a prospective observational multicenter study was undertaken for the validation process. We evaluated the measurement properties of the J-FIHOA among Japanese hand OA patients within the classical test theory framework, referring to the consensus-based standards for the selection of health measurement instruments (COSMIN) risk of bias checklist to scrutinize the methodological quality of our study [34].

### Translation and cultural adaptation

An expert committee was convened. It was comprised of two language professionals (A.I. and D.S.), three health professionals (Y.N., S.K. and H.H.), and an American native English-speaking research assistant (J.C.). Forward translation was performed independently by one professional translator who had no prior knowledge of the study and the two language professionals serving on the expert committee. All forward translators were native Japanese speakers and fluent in English. Results were assessed and synthesized into a preliminary version of the J-FIHOA. It was translated back into English independently by two English native professional translators, one with a medical background and one without. Both of them were blinded to the study aims and FIHOA concepts throughout the process. Additional forward and backward translations were undertaken to resolve specific points such as linguistic problems. We consulted with the FIHOA developers (E.M. and R.L.D.) about certain discrepancies and issues of interpretation. The translated FIHOA was pretested on 10 Japanese hand OA patients to identify potentially difficult words or phrases. We added kana script above difficult Chinese characters to facilitate comprehension, as is common in written Japanese. We also inserted a question and answer example illustrating how to mark responses. The committee submitted written reports to the developers that documented all processes and how we reached consensus. After their approval, the translation and cultural adaptation process was completed (Table 1).

**Table 1:**
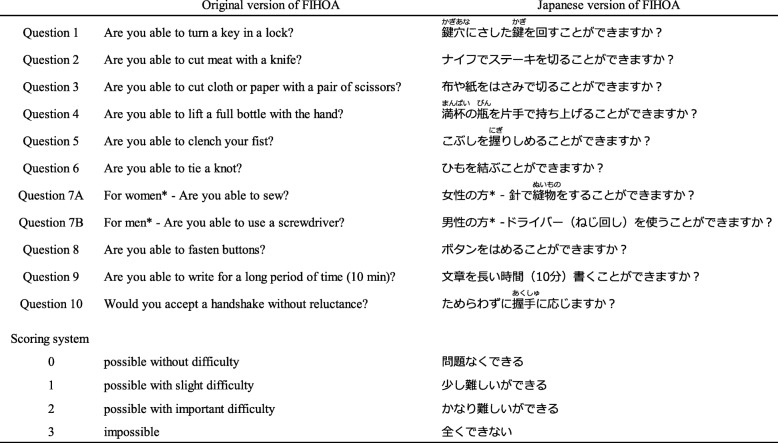
Original and Japanese versions of the Functional Index for Hand Osteoarthritis (FIHOA)

The FIHOA consists of 10 questions. Question 7 has two gender-role specific items requesting a separate response from female and male patients (Questions 7A and 7B, respectively). Patients score each item from 0 (possible without difficulty) to 3 (impossible) and the total score ranges from 0 to 30. The Japanese version has kana script above difficult Chinese characters to facilitate comprehension. *In this study, we removed “for women” and “for men” from Question 7 and asked all patients to answer both items to obtain all 11 responses regardless of gender

### Validation

#### Participant recruitment

Our university hospital and 16 other hospitals recruited hand OA patients at the outpatient departments from September 2017 to December 2018. New or already followed hand OA patients who were Japanese natives and over 20 years old were eligible. American College of Rheumatology (ACR) classification criteria for hand OA was used for the diagnosis [35]. Patients with other rheumatic diseases or post-traumatic OA were excluded. Participants had conventional therapies for hand OA. Written consent was obtained from each participant. The study protocol was approved by the review board of each participating hospital.

#### Data collection

At the enrollment visit a medical history (including duration of hand pain/stiffness and previous treatment for hand OA), postero-anterior radiographs of both hands and the following patient reported questionnaires were collected: J-FIHOA, Hand20, Japanese version of the Stanford Health Assessment Questionnaire (HAQ), numerical rating scale for pain (NRS pain), and Japanese version of the Short Form 36 Health Survey (SF-36).

Participants were followed up to 1 year to collect longitudinal data sets. For the test-retest reliability, we obtained J-FIHOA data from those whose symptoms and treatment were unchanged over at least 3 months to avoid disease flares or therapeutic modifications. The test-retest interval was one to 2 weeks. Examinees were allowed to answer the retests either at a face-to-face visit or, for their convenience, via postal mail. To assess the responsiveness, we selected symptomatic hand OA participants who started or changed to certain new systematic pharmacological treatments limited to oral acetaminophen, NSAIDs and/or tramadol. We checked J-FIHOA and other questionnaire scores immediately before the treatment and at a 4-week follow-up visit (+/− 2 weeks). At the follow-up visit, these participants also evaluated the change in the clinical state of their hands using a 7-point Likert scale (global rating of change [GRC]).

#### Questionnaires

##### J-FIHOA

The FIHOA consists of 10 questions, one of which requests a separate response from females, *Are you able to sew?* and males, *Are you able to use a screwdriver?* (Question 7). In this study, we removed “for women” and “for men” from Question 7 to obtain all 11 responses regardless of gender. Participants answered all 11 items, from 0 (possible without difficulty) to 3 (impossible), and we calculated the J-FIHOA scores in two different ways. One was to sum the 10 items as the original FIHOA does, called the “total score” (range, 0 to 30). Participants with total scores of 5 or more were defined as having symptomatic hand OA. The other was to sum all 11 items, called the “11-item model.” We used the 11-item model only when performing exploratory factor analysis and pursuing internal consistency on Question 7.

##### Hand20

The Hand20 is composed of 20 illustrated questions for disorders that assess the upper limb including hands. Scoring for each item ranges from 0 to 10, higher numbers indicating greater disability. The total score is obtained by dividing the sum of all questions in half (range, 0 to 100). Explanatory illustrations and short, easy-to-understand questions facilitate good response rates especially among elderly people [36, 37].

##### Japanese version of the Stanford health assessment questionnaire (HAQ)

The HAQ is a widely used instrument to assess functional disability especially in RA [38]. Regarding cultural differences, 3 questions on the Japanese version of HAQ have been modified: *get in and out of bed* to *get up and down from futon*, *cut your meat* to *use chopsticks for meal*, and *a 5 pound object* to *a 2 l plastic bottle* [39]. Scores are increased to 2, if they were lower, in any categories in which the patient used a device or relied on help from another person.

##### Numerical rating scale for pain (NRS pain)

Global pain of the affected hand(s) was assessed using a numerical rating scale. Participants were asked to mark the level of their pain on a horizontal scale from “0 = no pain” to “10 = worst pain imaginable.” [40]

##### Japanese version of the short form 36 health survey (SF-36)

The SF-36 is a questionnaire to measure general health status with 36 questions consisting of eight scales that can be summarized into components. It has been validated in Japanese [41]. A three-component model is used when analyzing results of the Japanese version of SF-36 scores: physical component summary (PCS), mental component summary (MCS) and role-social component summary (RCS) [42].

##### Global rating of change (GRC) scale in clinical state of the hands

Global rating of change (GRC) scales are commonly used to evaluate the responsiveness and to calculate the minimal clinically important change of the scale [43]. We used a GRC scale for symptomatic hand OA patients who started or changed to the new pharmacological treatments. At the 4-week follow-up visit, patients were asked “How would you describe your hand condition compared to before you took the new drug?” and scored their change using a 7-point scale: very much improved, much improved, a little improved, no change, a little deteriorated, much deteriorated, or very much deteriorated. We categorized patients based on the GRC scale and performed subgroup analyses.

#### Data analysis

Descriptive analyses were performed to summarize patient characteristics and scores of questionnaires. Incomplete J-FIHOA items were also examined. We compared the characteristics between female and male participants using the Student’s *t-*tests for continuous variables and chi-square tests for categorical variables. By checking the distribution of each questionnaire with the Kolmogorov-Smirnov test, we found that the only measure normally distributed was the SF-36 MCS. We used the Mann-Whitney U test to examine gender difference in each item or total score, and the Spearman’s rank correlation coefficients to measure the strength of the associations among items and/or questionnaires. Correlations were categorized as none (*r* = 0–0.29), weak (*r* = 0.30–0.49), moderate (*r* = 0.50–0.69) or strong (*r* = 0.70–1.00). All analyses were carried out using IBM SPSS software, version 24. The level of significance was set at *p* values of less than 0.05.

#### Validation

##### Structural validity

Using the 11-item model, factor analysis was performed with the maximum likelihood method to explore the scale structure of the J-FIHOA. Since the original FIHOA is a hand OA-specific scale, the Japanese version was expected to be unidimensional. We determined the number of relevant factors based on eigenvalues larger than one (the Kaiser criterion) and visual inspection of the scree plot [44].

##### Internal consistency

Internal consistency was evaluated with Cronbach’s alphas and correlations between each individual item and the total score of J-FIHOA without it (item-total correlations). To examine gender difference, we compared the score of each item and performed an additional investigation to explore the measurement properties of Questions 7A and 7B, which consist of two gender-role specific items. Cronbach’s alphas and item-total correlations were re-calculated in the 11-item model.

##### Reliability

We selected participants in stable condition, whose symptoms and treatments were unchanged. They were asked to answer the J-FIHOA twice, repeating it after a one- to two-week interval. The intraclass correlation coefficient (ICC) was used to assess test-retest reliability.

##### Construct validity

To assess construct validity, we performed hypothesis testing by focusing on correlations between the J-FIHOA and the other validated scales. Six hypotheses were established prior to data collection: Hand20 correlation would be the strongest among instruments; HAQ and NRS pain correlations would be moderate; SF-36 PCS correlation would be moderate but MCS and RCS would be weak or none.

##### Responsiveness

To analyze responsiveness, we recruited symptomatic participants whose total J-FIHOA scores were 5 or more and who were starting oral analgesic drugs. We used scores immediately before the treatment and at the 4-week follow-up visit (+/− 2 weeks). We compared the scores using the Wilcoxon signed-rank test. The effect size (ES) and standardized response mean (SRM) were also evaluated. ES was obtained by dividing the mean change of scores by the standard deviation (SD) of initial scores. SRM was obtained by dividing the mean change of scores by the SD of that change.

We evaluated responsiveness using two different approaches: comparisons of ES and SRM between the J-FIHOA and other measurements; and subgroup analyses of the J-FIHOA. We expected the J-FIHOA to have the largest ES and SRM among all the PROMs, except for NRS pain. We used the GRC scale for subgroup analysis. At the end of data collection, thirty data sets were available for responsiveness analysis. Almost all patients reported their changes as either a little improved (*n* = 14) or much improved (*n* = 12) and there were no complaints of deterioration. The remaining patients said very much improved (*n* = 1) or no change (*n* = 3). So, we divided the patients into two subgroups based on the GRC scale: major change group (very much improved and much improved, *n* = 13) and minor change group (a little improved and no change, *n* = 17). We hypothesized that the major change group would have larger ES and SRM on the J-FIHOA than the minor change group.

## Results

### Translation

Most FIHOA items were deemed equivalent in the translation and cultural adaptation process but discrepancies between the original and back translation revealed some issues. On a few major issues, the committee consulted with the FIHOA developers (Additional file 1). Considering possible cultural differences, Question 10 *Would you accept a handshake without reluctance?* was actively debated. The committee decided to translate Question 10 close to the original and explore its rationale in the following validation process.

### Descriptive results at enrollment visit

A total of 178 female and 32 male hand OA patients participated (Table 2). Mean age at the enrollment visit was 64.6 years. Approximately 90% of participants had pain and more than 60% experienced stiffness. Mean duration of pain and stiffness were both 5.2 years. There were no statistically significant gender differences in the patient characteristics and PROMs. Mean total score of J-FIHOA was 5.5 (SD 5.8) and almost half of participants met the definition of symptomatic hand OA. Only two of the 210 participants (1.0%) failed to answer one or two J-FIHOA items. Questions 4, 7A, and 10 each had one missing response.

**Table 2:**
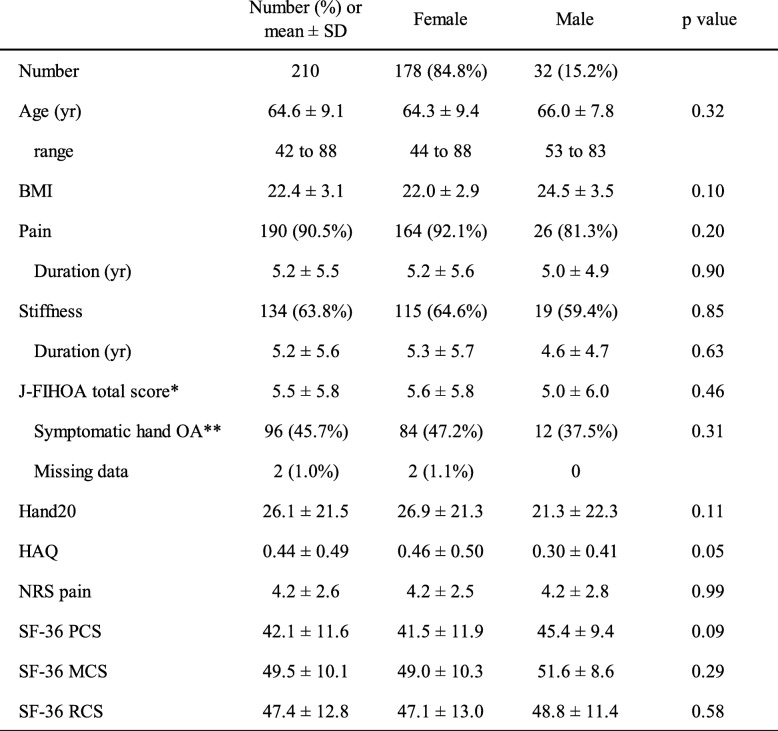
Characteristics and patient reported outcome measures (PROMs)

The values represent number (%) of participants or mean (SD) scores. Gender differences were evaluated using the Student’s t-tests for continuous variables or chi-square tests for categorical variables. *Sum of 10 items with a separate answer from female and male participants (Question 7). **Participants with J-FIHOA total scores of 5 or more. BMI: body mass index, NRS: numerical rating scale, PCS: physical component summary score, MCS: mental component summary score, RCS: rolesocial component summary score

### Unidimensionality

**Fig. 1:**
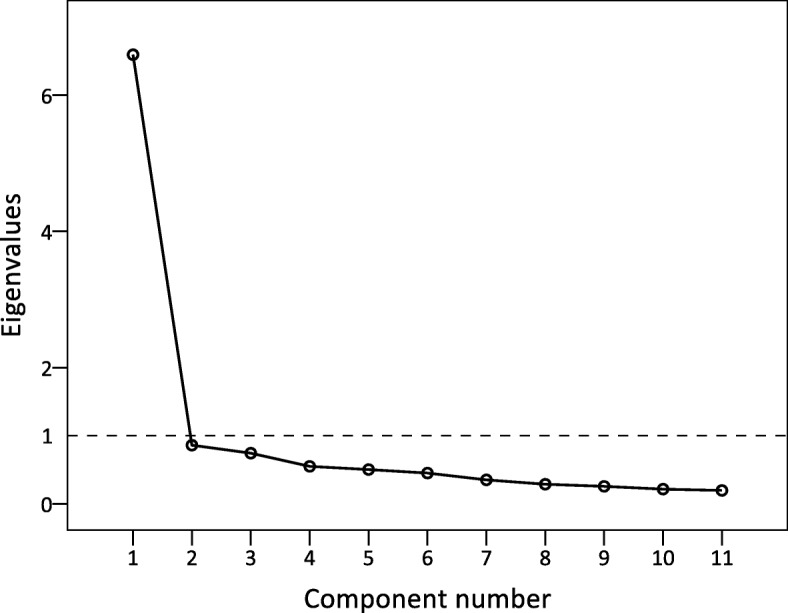
Scree plot of J-FIHOA. The eigenvalue for the first factor was greater than one and accounted for almost 60% of the total variance. The single elbow in the scree plot also indicated that the J-FIHOA was a unidimensional scale

The eigenvalue of the first factor was 6.59, which explained the 59.9% of the total variance in the J-FIHOA. Comparatively, the second factor was 0.86 accounting for only 7.8% of the variance. The scree plot had a single elbow curve (Fig. 1). These findings confirmed that the J-FIHOA had a unidimensional structure. We also examined the factor loadings, representing associations between each item and the factor, which ranged from 0.61 to 0.85.

### Internal consistency

Cronbach’s alpha among females was 0.914 with a 95% confidence interval (CI) from 0.893 to 0.931. Among males it was 0.929 (95% CI, 0.885 to 960). Item-total correlations were also obtained independently and the values ranged from 0.580 to 0.779 among females and from 0.508 to 0.881 among males (Table 3). No item scores had gender differences, except for Question 4. The item-total correlations of the 11-item model revealed that Question 7B *Are you able to use a screwdriver?*, originally *for men*, had the strongest correlation in female participants and vice versa (0.809 and 0.885, respectively).

**Table 3:**
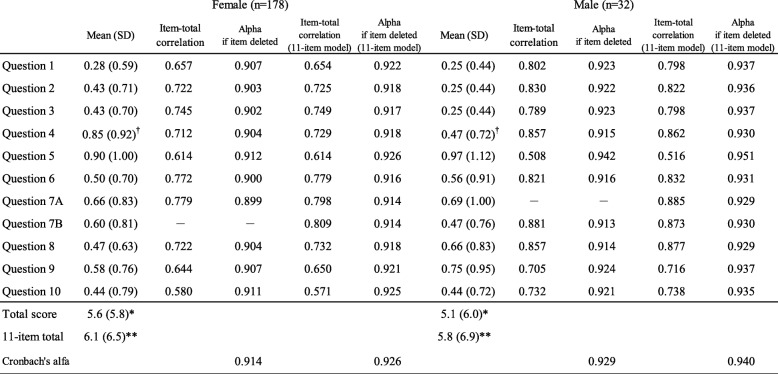
Item-total correlations

Item scores and item-total correlations of the J-FIHOA. Mean scores between female and male groups were compared using the Student’s t-test. Correlations are shown using both the total J-FIHOA and the 11-item model. *Total score of the J-FIHOA, the sum of 10 items with a separate answer from female and male participants (Question 7). **Total score of the 11-item model, the sum of all 11 items. †*p* < 0.05

### Test-retest reliability

One hundred thirty-five patients repeated the J-FIHOA at one- to two-week intervals when their clinical conditions were stable during the observation period. Their longitudinal data sets were used to evaluate test-retest reliability. Mean J-FIHOA scores were 4.8 (SD 5.7) at the test and 5.5 (SD 5.9) at the retest. The ICC was 0.83 (95% CI, 0.77 to 0.88).

### Construct validity

We performed hypothesis testing focusing on correlations between the J-FIHOA and the other six validated scales. Among the six prior hypotheses, four correlations fulfilled our expectations: Hand20 had the strongest correlation among all instruments (*r* = 0.82); NRS pain was moderately correlated (*r* = 0.58); and both mental and role-social components of SF-36 had no correlations (*r* = − 0.24 and − 0.26, respectively). HAQ had a stronger correlation (*r* = 0.73) and the physical component of SF-36 had a weaker correlation (*r* = − 0.36) than our assumptions (Table 4).

**Table 4:**
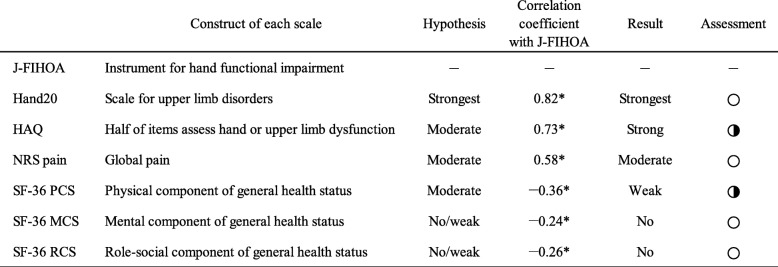
Hypothesis and correlations between J-FIHOA and other questionnaires

Hypotheses were based on these fundamental assumptions: the FIHOA scores reflected the severity of physical dysfunction of the hands and the Japanese version was equivalent to the validated versions of the FIHOA. Correlations were shown between the J-FIHOA and other validated PROMs: Hand20, HAQ, NRS pain and each component summary score of SF-36. *p < 0.05, Spearman’s rank correlation. PCS: physical component summary score, MCS: mental component summary score, RCS: role-social component summary score. ○: met our expectation, ◑: failed to meet our expectation but consistent with construct of the FIHOA, ●: disproved the hypothesis

### Responsiveness

Thirty symptomatic patients started new pharmacological treatments during the observation period. They had a 1-month follow-up visit and completed the questionnaires in both pre- and post-treatment conditions. Their longitudinal data sets were pooled to evaluate responsiveness. Before treatment, the mean J-FIHOA score was 11.6 (SD 4.7) and NRS pain was 6.8 (SD 1.8). After oral pharmacological treatments, J-FIHOA and NRS pain decreased by 3.2 and 2.0, respectively. ES and SRM of the J-FIHOA were − 0.68 and − 0.62. NRS pain showed a larger ES and SRM than the J-FIHOA did, while smaller ES and SRM were observed on the Hand20 and HAQ (Table 5). We performed subgroup analysis based on the GRC scale. J-FIHOA scores decreased by 4.5 in the major change group and 2.2 in the minor change group. The major change group demonstrated a larger ES and SRM.

**Table 5:**
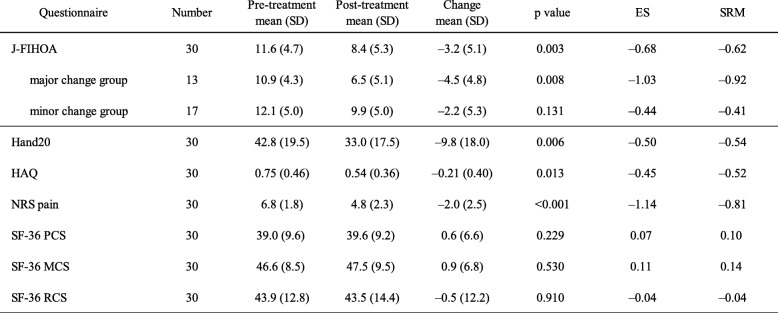
Responsiveness of J-FIHOA and other questionnaires

Pre- and post-treatment data sets were used to assess the responsiveness. *P* values were calculated using the Wilcoxon signed-rank test. ES was obtained by dividing the mean change scores by the standard deviation of the scores at pre-treatment. SRM was obtained by dividing the mean change of scores by the SD of that change. Patients who scored their change “very much improved” or “much improved” were categorized into the major change group and the others into the minor change group. The J-FIHOA showed the largest ES and SRM among all questionnaires, except for NRS pain. The major change group had a larger ES and SRM on the J-FIHOA than the minor change group. ES: effect size, SRM: standardized response mean

## Discussion

We created the J-FIHOA through a process of forward and backward translations to address the linguistic and cultural differences. We then performed a prospective multicenter study to validate its measurement properties with 210 Japanese hand OA patients with widely ranging conditions from well-controlled to severely symptomatic.

The translation process was performed following guidelines and publications distilled from comparable initiatives [22, 23]. Justifications for cultural adaptation depend on concepts of the instrument and populations concerned. Although the FIHOA consists of simple questions focusing on daily activities, several points needed to be scrutinized, especially the experimental equivalence of Question 10 *Would you accept a handshake without reluctance?*. As a cultural convention, Japanese, the elderly in particular, shake hands infrequently. Yet, the committee and developers agreed that Question 10 was unique and irreplaceable because it assessed aspects of aesthetics and communication.

Exploratory factor analysis revealed that the J-FIHOA was a unidimensional scale and all loadings satisfied the minimum requirement, usually set at 0.5 [44]. All questions contributed to a single factor, which we considered representative of “functional impairment.” Cronbach’s alphas were above 0.9 and each item-total correlation exceeded 0.5. These findings indicated that the J-FIHOA was a unidimensional scale with good internal consistency [45]. Question 10 also showed feasibility and consistency with only one missing response.

We also examined another concern regarding gender differences using the 11-item model [32]. Females had a statistically higher score (i.e. greater disability) in Question 4 *Are you able to lift a full bottle with the hand?*. We assumed it might be due to inherent differences, such as in muscle strength [46]. Both Questions 7A and 7B had the strongest item-total correlations (i.e. 7A *for women*, 0.885 among males and 7B *for men*, 0.809 among females), suggesting that the gender specific parts were irrelevant and could be removed. This gave rise to another concern regarding how to treat Question 7—measure both items, delete either, or combine. We were indecisive but disinclined to deviate from the original and kept “for women” and “for men” in the J-FIHOA. Additional investigation might be necessary such as differential item functioning (DIF) analysis, which assesses different probabilities of responding to certain items among different groups (ideally with more than 100 patients per group) [34].

Several validated PROMs were used to examine associations with the J-FIHOA. In addition to the FIHOA, the Australian/Canadian Hand OA Index (AUSCAN) is the other hand OA-specific assessment tool [47]. AUSCAN has excellent measurement properties and has been frequently applied to hand OA clinical trials [26, 27, 32]. Although a number of linguistic versions are available, AUSCAN has not been translated and validated into Japanese. In addition, the tool is not freely available. Therefore, we did not include AUSCAN in this study.

Construct validity was assessed by testing hypotheses based on the fundamental assumptions that the FIHOA scores reflected the severity of functional impairment of the hands and that the Japanese version was equivalent to validated versions of the FIHOA. Hand20 assesses upper limb dysfunction and has some similar items to the J-FIHOA such as “Do up shirt buttons with both hands” (cf. Question 8 *Are you able to fasten buttons?*). It showed the strongest correlations (r = 0.82), as expected. Although the FIHOA has no pain-related items, pain has been reported to be moderately correlated on the FIHOA and our data were consistent (*r* = 0.58) [24–26, 28–31]. Since mental and social status are dimensions distinct from physical condition, no correlations were observed in the SF-36 MCS and RCS (*r* = − 0.22 and − 0.23, respectively). Our results showed that the HAQ had a stronger correlation (*r* = 0.73) and SF-36 PCS had a weaker correlation (*r* = − 0.36) than previous reports, where both HAQ and SF-36 PCS were moderately correlated with the FIHOA (r = 0.57 to 0.73 and *r* = − 0.57 to − 0.67, respectively) [26, 29–31]. We concluded that our results were not inconsistent with the construct of the J-FIHOA because these correlations largely depended on patient characteristics or conditions.

Longitudinal data enabled an evaluation of the test-retest reliability (135 participants) and responsiveness (30 participants). In the test-retest analysis, we allowed the examinees to answer the retests either at a face-to-face visit or via postal mail. Most chose the latter. Although the different forms of administration might have affected the reliability, the ICC was 0.83. Even the lower bound of the 95% CI was greater than 0.70, the minimal requirement of reliability, indicating the J-FIHOA had good test-retest reliability [44].

ES and SRM are widely used to evaluate responsiveness. However, the COSMIN suggests that both are inappropriate in some situations. One reason is that ES and SRM are highly dependent on the SD of initial scores and change scores, respectively. If the target population is homogeneous or the variation in treatment effect is small, these values can be large. So, we assessed responsiveness of the J-FIHOA by two construct approaches, comparing with other PROMs and dividing the patients into two subgroups [34]. The J-FIHOA showed the highest ES and SRM among all PROMs, except for NRS pain. We assumed that it was because J-FIHOA was a hand OA-specific scale and could detect subtle differences in function of the hands. It was unsurprising that NRS pain showed the best responsiveness among the questionnaires. We used oral analgesic drugs with a short duration for assessing responsiveness because no disease-modifying drugs are available and evidence does not support surgical intervention, especially for osteoarthritis of interphalangeal joints [48]. Previous clinical trials also revealed that pain scoring is the most sensitive tool in hand OA symptom assessment [49]. Subgroup analysis was performed by clustering the patients into two groups based on the GRC scale. As expected, the patients who reported greater improvements obtained a larger ES and SRM. Although the results indicated that the J-FIHOA had good responsiveness, the number of longitudinal data sets were relatively small (*n* = 30) and inadequate for further analyses such as of minimal clinically important difference [50].

The strengths of this study were the number and diversity of our hand OA patient panels, from well-controlled to awaiting scheduled surgery with severe symptoms. They enabled us to estimate precise internal consistency and easily generalize our results to a wide range of clinical and research settings. To our knowledge, with its 17 participating university and community hospitals, this is the first multicenter study conducted for hand OA research in Japan. We are confident that this framework will function effectively in future clinical investigations.

This study has several limitations. We did not evaluate measurement invariance by comparing Japanese and Western patients directly. Some cultural inequalities, such as in daily activity, personality trait and perception of functional impairment, may variously have influenced responses to each question. Data from two populations and DIF analysis would reveal differences at item levels. Another limitation is that we have not verified the diagnostic cut-off value of the J-FIHOA for defining symptomatic hand OA among Japanese patients. We neither recruited healthy individuals nor clearly defined non-symptomatic hand OA based on criteria other than the J-FIHOA scores. We anticipate future international trials or Japanese cohort studies will accumulate more evidence regarding the measurement properties of the J-FIHOA.

## Conclusions

We created the cross-culturally adapted J-FIHOA and validated it among Japanese hand OA patients. Our results show the equivalence with the original version and its advantageous measurement properties for assessing hand OA-related functional impairment. The J-FIHOA has the potential both to benefit clinical practice and to accelerate clinical research and therapeutic trials, by promoting comparisons and syntheses of studies within and outside of Japan.

## Supplementary information

**Additional file 1.** Summary points for translation and cultural adaptation.

## Data Availability

The datasets used during the current study are available from the corresponding author on reasonable request.

## Abbreviations

ACR: American College of Rheumatology
AUSCAN: Australian/Canadian Hand OA Index
BMI: Body mass index
CI: Confidence interval
COSMIN: Consensus-based standards for the selection of health measurement instruments
DIF: Differential item functioning
ES: Effect size
ESCEO: European Society on Clinical and Economic Aspects of Osteoporosis, Osteoarthritis and Musculoskeletal Diseases
FIHOA: Functional Index for Hand Osteoarthritis
GRC: Global rating of change
HAQ: Health Assessment Questionnaire
ICC: Intraclass correlation coefficient
J-FIHOA: Japanese version of the Functional Index for Hand Osteoarthritis
MCS: Mental component summary
NRS: Numerical rating scale
OA: Osteoarthritis
OARSI: Osteoarthritis Research Society International
PCS: Physical component summary
PROM: Patient reported outcome measure
RA: Rheumatoid arthritis
RCS: Role-social component summary
SD: Standard deviation
SF-36: Short Form 36 Health Survey
SRM: Standardized response mean
