# Supplementary material for: Cross-cultural translation, adaptation and validation of a Japanese version of the functional index for hand osteoarthritis (J-FIHOA)
Source: BMC Musculoskelet Disord. 2020 Mar 16;21:173. doi: 10.1186/s12891-020-03193-6 (PMC7333425; doi:10.1186/s12891-020-03193-6)
Supplement: Supplementary file 1 — Additional file 1. Summary points for translation and cultural adaptation. [file 12891_2020_3193_MOESM1_ESM.pdf]

Supplement A. Summary points for translation and cultural adaptation

**Question 2:**<br>Points<br>— "Are you able to cut meat with a knife?" evoked an eating or cooking situation.<br>— Other PROMs such as the J-HAQ performed cultural adaption regarding difference of eating utensils (chopsticks).<br>— Whether we should perform an adaptation was discussed.<br>Reasons<br>— Japanese contemporary eating style has assimilated fork and knife use.<br>— Question 2 evaluated the ability to grasp an object and hold it with enough strength.<br>— Chopsticks use might focus on other functions such as dexterity.<br>Consensus<br>— The committee decided not to alter the original question because the FIHOA is an instrument to assess hand function.
**Question 4:**<br>Points<br>— Japanese use neither definite/indefinite articles nor singular/plural forms and "with the hand" needed to be modified.<br>— The committee assumed that "the hand" should refer specifically to the more symptomatic side.<br>— "The hand" was translated to "waruihou no te" in the Japanese version, meaning the "one affected" hand.<br>Reasons<br>— Through discussion with the developers, the committee identified misunderstandings.<br>— Hand OA patients usually suffer bilaterally and this question assesses the ability of the hand that is used daily.<br>— Our preliminary version indicated usage of the more severely affected side and it could make the FIHOA score higher (i.e. worse function).<br>Consensus<br>— The committee revised "waruihou no te" to "kata te" (with "one" hand)
**Question 10:**<br>Points<br>— The committee needed to discriminate between the meaning of auxiliary verbs ("would you" though the other questions use "could you").<br>— Japanese, especially the elderly, shake hands less frequently.<br>Reasons<br>— The meaning of shaking hands is equivalent between Asian and Western countries.<br>— This question assessed not only hand function but also some aspects of aesthetics and/or communication.<br>Consensus<br>— The committee decided to translate it closely to the original and explored its reliability in the following validation process.
